# Long-term changes of parvalbumin- and somatostatin-positive interneurons of the primary motor cortex after chronic social defeat stress depend on individual stress-vulnerability

**DOI:** 10.3389/fpsyt.2022.946719

**Published:** 2022-07-28

**Authors:** Maria L. Serradas, Valentin Stein, Anne-Kathrin Gellner

**Affiliations:** ^1^Institute of Physiology II, Medical Faculty, University of Bonn, Bonn, Germany; ^2^Department of Psychiatry and Psychotherapy, University Hospital Bonn, Bonn, Germany

**Keywords:** chronic stress, parvalbumin, motor learning, depression, somatostatin, interneuron, motor cortex

## Abstract

Chronic stress is a major risk factor for developing mental illnesses and cognitive deficiencies although stress-susceptibility varies individually. In a recent study, we established the connection between chronic social defeat stress (CSDS) and impaired motor learning abilities accompanied by chronically disturbed structural neuroplasticity in the primary motor cortex (M1) of mice. In this study, we further investigated the long-term effects of CSDS exposure on M1, focusing on the interneuronal cell population. We used repeated CSDS to elicit effects across behavioral, endocrinological, and metabolic parameters in mice. Susceptible and resilient phenotypes were discriminated by symptom load and motor learning abilities were assessed on the rotarod. Structural changes in interneuronal circuits of M1 were studied by immunohistochemistry using parvalbumin (PV+) and somatostatin (SST+) markers. Stress-susceptible mice had a blunted stress hormone response and impaired motor learning skills. These mice presented reduced numbers of both interneuron populations in M1 with layer-dependent distribution, while alterations in cell size and immunoreactivity were found in both susceptible and resilient individuals. These results, together with our previous data, suggest that stress-induced cell loss and degeneration of the GABAergic interneuronal network of M1 could underlay impaired motor learning, due to their role in controlling the excitatory output and spine dynamics of principal neurons required for this task. Our study further highlights the importance of long-term outcomes of chronically stressed individuals which are translationally important due to the long timecourses of stress-induced neuropsychiatric disorders.

## Introduction

Stress represents a major risk factor for developing mental illnesses and cognitive deficiencies in humans (1–3). Aiding neurobiological research, chronic social stress has a strong impact on affective-like behavioral responses, inducing a robust depression-like phenotype marked by anhedonia, anxiety, and social-avoidance in mice (4–6). In recent years, stress research has focused on the impact of chronic social stress in the neural circuits of limbic and prefrontal areas of the brain, due to their implications in behavior, emotions, cognition and memory. However, less attention has been given to the motor cortex and its structure and function in the context of chronic stress. The primary motor cortex (M1) is considered to be a major region for initiating and controlling voluntary movements (7, 8) and has an imperative role in contributing to motor learning (9, 10), which can be severely affected by chronic social stress. In a recent study, we have established the connection between chronic social defeat stress (CSDS) and impaired motor learning abilities accompanied by chronically disturbed structural neuroplasticity in the primary motor cortex of mice. Strikingly, long lasting cellular alterations on the level of glial cells of the motor cortex and the surrounding cerebrospinal fluid were still observed 5 weeks after cessation of the stressor (6). These findings, which were dependent on the individual stress vulnerability of the mice, open the question of whether other cellular components like interneurons of the motor cortex can get affected under chronic social stress exposure.

The motor cortex contains a vast collection of cellular components, organized by layers, including many different types of interneurons (11). The interneuronal network consists mostly of GABAergic inhibitory connections and is necessary to control output activity of principal neurons, cells that have been investigated in our previous work (6). Previous studies of limbic brain regions suggest that the interneural network is one of the most affected structures by chronic stress (12–15). GABAergic dysfunction and disturbed inhibitory/excitatory balance have been found in neuropsychiatric disorders and preclinical models, mostly investigated in the prefrontal brain regions [reviewed in (16, 17)]. The calcium-binding protein parvalbumin (PV), and the neuropeptide somatostatin (SST) define the most predominant interneuron subtypes within the motor cortex, which together comprise approximately 70% of the total GABAergic cortical interneuron population (18, 19) that control intra- and intercortical output. PV+ interneurons, contact the soma and proximal dendrites or the initial axon segment of glutamatergic pyramidal cells (19). SST+ interneurons arborize into the dendritic tuft in layer I and modulate spine dynamics (18–20). These interactions are important for intact neuroplasticity in M1 which we recently showed to be severely affected by CSDS (6).

In this study, repeated CSDS was used to induce effects depending on individual stress vulnerability across behavioral, endocrinological, and metabolic endpoints in C57BL/6J mice. Motor learning skills were assessed on the accelerating rotarod and the motor cortex studied histologically for structural changes in interneuronal circuits of M1, paying special attention to the GABAergic inhibitory network by using PV and SST markers.

## Materials and methods

### Animals

Twenty-four adult male mice (C57BL/6J, age 11 ± 0.75 weeks) were single housed throughout the entire experiment except for the stress period. Mice were fed *ad libitum*, maintained under a 12-h light-dark cycle and constant room temperature (22°C). Mice were weighed daily during the stress phase, the behavioral experiments and before tissue collection. All experiments were performed following the guidelines of the German Animal Protection Law and Directive 2010/63/EU of the European Commission and have been approved by the government of North Rhine Westphalia (Local Committee for Animal Health, LANUV NRW). Animal experiments have been reported in compliance with the ARRIVE guidelines.

### Chronic social defeat stress

Mice were randomly assigned to either stress or control treatment with a ratio of 1.4:1 for group size. This ratio is necessary to generate sufficient numbers of the less frequently occurring resilient mice (4, 6) and avoid underpowering the analysis, in compliance with the 3R of animal research. CSDS was applied as described by Golden et al., (4). For 10 consecutive days, the experimental mouse was introduced to the home cage of an unknown, bigger and aggressive CD1 mouse (aggressor) for 5 min, where it encountered several physical attacks and threats. Afterward, both the stressed mouse and CD1 remained in the same cage for 24 h, separated by a perforated acrylic glass divider allowing continuous sensory cues. Control mice were handled daily and housed pairwise in an equally divided cage. Pairings and cages were not changed throughout the CSDS period. One mouse of the CSDS group died after 7 days without any apparent reason (such as wounding during attacks or sickness behavior) and was excluded from the analysis. In the CSDS group, the daily 5 min of physical exposure to the aggressor were recorded on video for *post-hoc* analysis of attacks. In 8 cases single sessions (randomly occurring during the 10 days and in different mice) were not recorded due to technical failure. Each attack of the CD1 toward the experimental mouse was counted and rated with a severity score from 1 (short physical contact without bite) to 3 (biting and full body contact including pinning to the ground) in score intervals of 0.5 (allowing for more nuanced scoring in cases where an attack consists of a behavioral mix).

### Behavioral tests

#### Nestlet shredding test

Nest shredding analysis was performed as described by Deacon (21). Briefly, old nesting material was removed from the home cage. A new nestlet was placed into the animal’s cage. After 3 h, the nest-building performance of the mouse was assessed on a rating scale of 1 to 5 (1 = nestlet untouched, 5 full nest and all material used).

#### Sucrose preference test

Mice were habituated to the smaller bottles during the CSDS phase. After the last CSDS session, all mice were single housed and received two bottles, one filled with water and one with 1% sucrose solution. Position of the bottles was switched after 24 h. Consumption of water and sucrose solution was measured by weighing the bottles at 0, after 24, and 48 h. Sucrose preference was calculated as the relative consumption of sucrose solution and averaged between the first and second day of the test.

#### Social avoidance test

Approximately 24 h after the last CSDS session each experimental mouse was placed in an open arena (40 cm × 40 cm) together with an empty wire cage and was left to explore for 2.5 min, which were recorded on video. The mouse was removed from the arena and the empty wire cage replaced by one filled with an unknown CD1 mouse. Exploration of the experimental mouse was again recorded for 2.5 min. Both trials were then analyzed using Anymaze software (Stoelting) and the number of entries of the mouse head interacting with the wire cage within a 4 cm zone (with and without presence of a social partner) was calculated.

### Accelerating rotarod test

Mice were first habituated by placing them onto the rod at slow speed (4 rpm) until a calm and steady movement was observed for a minimum of 180 s. The motor learning was tested by accelerating the rotation speed from 4 to 20 rpm (increment of 1 rpm/s for 16 s, then remaining at 20 rpm for 74 s) for each trial. Time until the animal fell (or cut-off time of 90 s) was recorded for 15 consecutive trials and mice were allowed to rest for 60 s in-between trials. Learning curves were fitted by a sigmoid curve derived from the Hill equation and maximum time on the rod calculated.

### Fecal corticosterone ELISA

All mice were moved to a fresh home cage (single-housed) after finishing the last session of CSDS or control handling. After 24 h, fecal pellets were collected from the bedding and stored at −20°C until further processing. Corticosterone (CORT) levels in feces were determined by an ELISA kit according to the manufacturer’s instructions (Arbor Assays, K014- H5). All samples and standards (78.128–10,000 pg/ml) were tested in duplicates. Two samples from the control group had to be excluded due to a technical error.

### Brain tissue collection

Mice were deeply anesthetized (Ketamine 240 mg/kg and Xylazine 32 mg/kg body weight) until complete loss of reflexes before transcardiac perfusion with 50 ml cold phosphate-buffered saline (PBS, pH 7.4) followed by 50 ml paraformaldehyde (4% in PBS, pH 7.4). The brain was removed and transferred to ice-cold paraformaldehyde (4% in PBS, pH 7.4) overnight before dehydration in sucrose (30% in PBS, pH 7.4), freezing in isopentane on dry ice and storage at −80°C until further processing.

### Immunohistochemistry

Coronal sections of 40 μm thickness were cut through the motor cortex on a cryostat (Leica) and stored in antifreeze solution at −20°C until further processing. Sections were washed in PBS (pH 7.4) 3 × 10 min before blocking for 30 min in 0.1% Tween20 (Sigma) solution with 3% normal goat serum (Gibco). Incubation for 24 h at 4°C with primary antibodies (anti-PV 1:250, abcam ab11427; anti-SST 1:300, BMA T-4103) in blocking solution containing additional 5% bovine serum albumin (Sigma) followed. After another washing step (3 × 10 min), sections were incubated with a corresponding secondary antibody (Alexa Fluor^®^ 568, Invitrogen A-11011) at 1:500 in 0.5% Tween20 solution for 1 h at room temperature. Specificity of both primary antibodies for their targets had already been determined elsewhere (22). As a negative secondary antibody control, randomly selected sections from different mice of the cohort were stained simultaneously using the same protocol except for the primary antibody. After washing for 3 × 20 min in PBS pH 7.4 including a nuclear counterstaining with DAPI (4′,6-diamidino-2-phenylindole 1:500, abcam) sections were mounted on slides and protected by Fluoro-Gel mounting medium (EMS).

### Image acquisition

Sections containing the primary motor cortex at Bregma AP 1.7 to 0.7 mm according to Franklin and Paxinos (23) were imaged with a fluorescence microscope (BZ-X800, Keyence) equipped with a 10× objective, a DAPI and Texas Red filter cube for the DAPI and Alexa Fluor^®^ 568 signal, respectively. Using the BZ-X800 Viewer software package, images through the entire section were acquired as stitched z-stacks with a step size of 1 μm at a xy-resolution of 1.5 μm/px. Acquisition settings were kept constant for all samples within each staining. Images were saved at the best focus plane as determined by the BZ-X800 Viewer software package.

### Cell quantification, size, and intensity measurements

For all image processing and analysis, Fiji (24) was used. The area of the primary motor cortex (M1) was manually outlined according to Paxinos and Franklin (23). The layers of M1 were delimited with the aid of the Scalable Brain Atlas (25) in the DAPI channel. Images were individually thresholded based on their background in the red channel (containing the interneuronal staining) and the watershed algorithm applied to separate potential overlapping cells. Subsequently, cells located within M1 and its layers were counted using the Particle Analyzer plugin. A minimum particle size of 40 μm^2^ was established and set to avoid the counting of artifacts. Size and integrated density [representing the cumulatively available PV or SST content in the cells; expressed in arbitrary units (A.U.)] were measured for each counted particle. We corrected density measurements for background fluorescence for all particles in each section individually (integrated density–background × particle area).

### Statistical analysis

**FIGURE 1:**
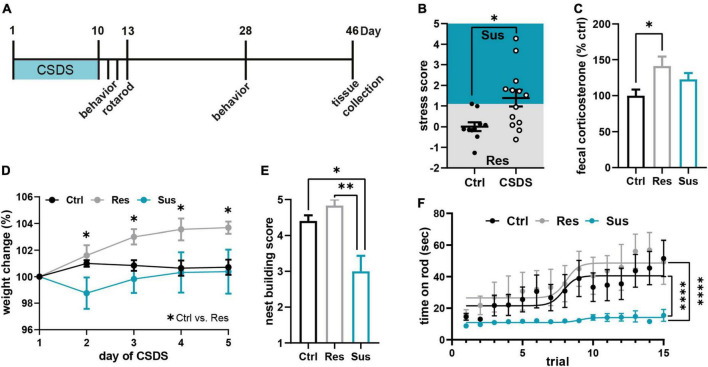
Chronic social defeat stress (CSDS) induces severe and lasting behavioral and physiological changes. **(A)** Experimental timeline. **(B)** Stress score derived from three behavioral tests [sucrose preference test (SPT), nestlet shredding test (NST), social avoidance test (SAT); Supplementary Figure 1] classified mice as stress susceptible or resilient. **(C)** Cumulative fecal corticosterone (CORT) levels from 24 h post CSDS normalized to the control group. **(D)** Weight development of the three behavioral groups during the first 5 days of CSDS. **(E)** Chronic stress-induced changes in the nest building test on day 28, 18 days post CSDS. **(F)** Motor learning assessed by the accelerating rotarod task 3 days post CSDS. *P < 0.05, ^**^P < 0.01, ^****^P < 0.0001. Results are shown as mean ± SEM.

**FIGURE 2:**
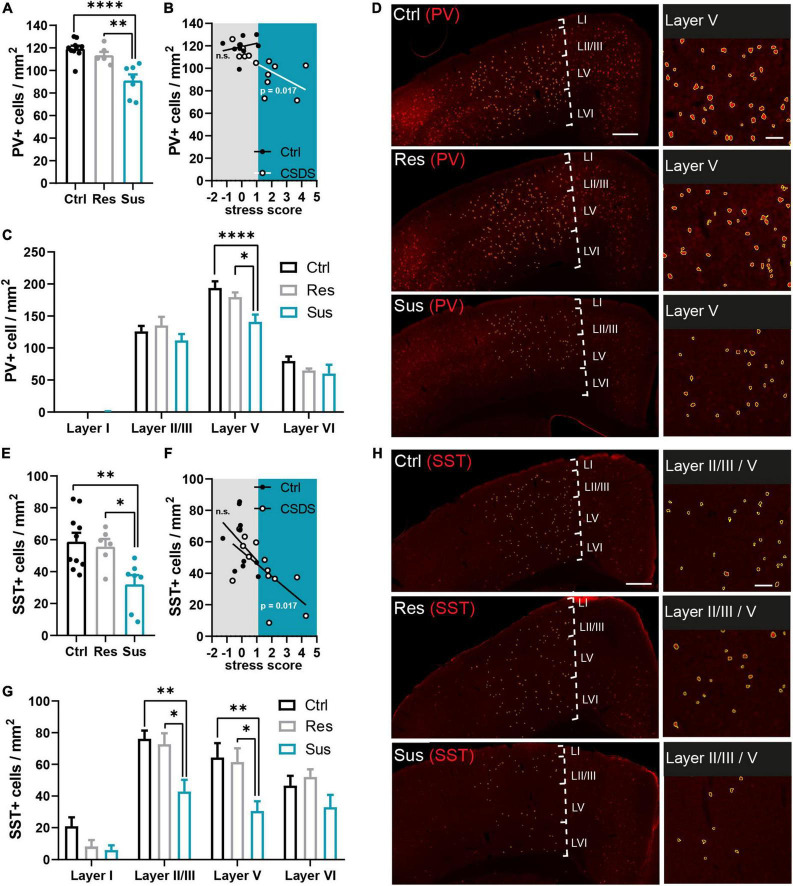
Interneuron populations are reduced in the primary motor cortex long-term after CSDS depending on stress vulnerability. **(A)** Parvalbumin (PV+) cell density in the analyzed M1 area compared between the behavioral groups. **(B)** Predictive relationship of the stress score and PV+ cell density in M1 analyzed for the stressed and unstressed group (gray area: stress score spectrum of controls and resilient mice, blue area: stress score spectrum of susceptible mice). **(C)** Layer-wise assessment of the stress effect on PV+ cell density in M1. **(D)** Examples of the PV+ cells (red = PV+ immunostaining, yellow = outlines of PV+ cells identified *via* semi-automated image analysis) in M1 and affected layers. **(E)** Somatostatin (SST+) cell density in the analyzed M1 area compared between the behavioral groups. **(F)** Predictive relationship of the stress score and SST+ cell density in M1 analyzed for the stressed and unstressed group (gray area: stress score spectrum of controls and resilient mice, blue area: stress score spectrum of susceptible mice). **(G)** Layer-wise assessment of the stress effect on SST+ cell density in M1. **(H)** Examples of the SST+ cells (red = SST+ immunostaining, yellow = outlines of SST+ cells identified *via* semi-automated image analysis yellow outlines) in M1 and affected layers. *P < 0.05, ^**^P < 0.01, ^****^P < 0.0001. Results are shown as mean ± SEM. Left: scale bar = 500 μm, right: scale bar = 100 μm.

**FIGURE 3:**
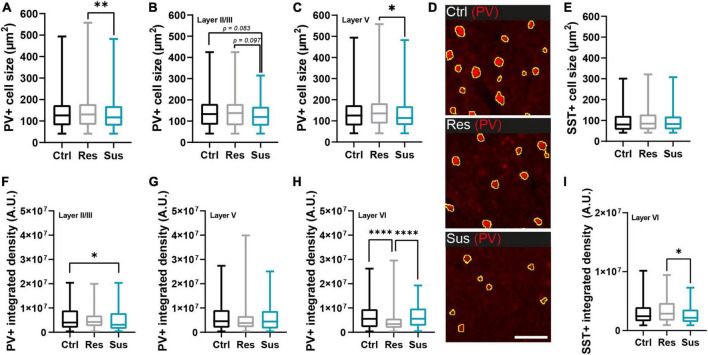
Interneuron populations are morphologically changed in the primary motor cortex (M1) long-term after CSDS depending on stress vulnerability. **(A)** Cell size changes of parvalbumin positive (PV+) cells in M1 and **(B,C)** its layers II/III and V (unchanged layer VI in Supplementary Figure 3). **(D)** Examples of PV+ cells from layer V in the different behavioral groups (scale bar = 100 μm). **(E)** Cell size of somatostatin positive (SST+) cells in M1. **(F–H)** Integrated density expressed as alterations of PV+ cells in the different M1 layers. **(I)** Intensity alterations of SST+ cells in layer VI (unchanged layers II/III and V in Supplementary Figure 3). *P < 0.05, ^**^P < 0.01, ^****^P < 0.0001. Results are shown as median, 25th and 75th percentiles, and minimum/maximum.

All behavioral tests, motor learning, microscopy, and image analysis were conducted by experimenters blinded for the treatment. The target number of mice used for the experiment was determined based on numbers in previously published studies and our experience with the model. Statistical analyses were performed in Graphpad Prism Version 8.0.1. The statistical test and group sizes are indicated in the results text. Data are presented as mean ± SEM in Figures 1, 2, as median, 25th and 75th percentiles, and minimum/maximum in Figure 3. Validity of the statistical approach was ensured by testing all data distributions for normality (D’Agostini-Pearson test). Depending on the outcome parametric or non-parametric tests were used for group comparisons. For repeated measures ANOVA, Greenhouse-Geisser correction was applied. Significance was assumed at alpha = 0.05, with two-sided testing. Tukey’s or Dunn’s *post-hoc* tests were applied in case of multiple comparisons after ANOVA or Kruskal-Wallis test, respectively.

## Results

### Chronic social defeat stress changes affective and motor learning behavior

All mice were subjected to 10 days of CSDS, or control handling and stress-induced behavior was assessed within 2 days post stress (experimental timeline in Figure 1A). The average stress score based on this behavioral assessment [sucrose preference test (SPT), nestlet shredding test (NST), social avoidance test (SAT), Supplementary Figure 1] showed a significant increase of stressed behavior in the group of mice exposed to CSDS (t_21_ = 2.501, *P* = 0.021, Student’s *t*-test, Ctrl *n* = 10, CSDS = 13 mice; Figure 1B). Each animal was classified as susceptible or resilient based on its deviation of the score from the control group. There was no difference in attack quantity and severity encountered by the resilient and susceptible subgroup during CSDS (Supplementary Figure 2). Cumulative CORT levels in the feces from the 24 h period after the last stress session corroborated the behavioral classification and revealed a significant increase of CORT release in resilient mice compared to controls while susceptible mice did not show this elevation post stress [*F*_(2_,_18)_ = 4.309, *P* = 0.030, one-way ANOVA with Tukey’s *post-hoc* test, Ctrl n = 8, Res *n* = 6, Sus *n* = 7 mice; Figure 1C]. The two stress phenotypes had markedly different weight developments throughout the first 5 days of CSDS: resilient mice gained weight compared to control and susceptible mice [time *F*_(4_,_80)_ = 4.713, *P* = 0.002; stress *F*_(2_,_20)_ = 3.896, *P* = 0.037; interaction *F*_(8_,_80)_ = 2.404, *P* = 0.022, RM ANOVA with Tukey’s *post-hoc* test, Ctrl *n* = 10, Res *n* = 6, Sus *n* = 7 mice; Figure 1D]. Persistence of the stress-induced phenotypes was confirmed at day 28 by a significantly reduced nest building score in susceptible mice vs. control and resilient mice (H_2_ = 11.29, *P* = 0.004, Kruskal-Wallis test with Dunn’s *post-hoc* test, Ctrl *n* = 10, Res *n* = 6, Sus *n* = 7 mice, Figure 1E) as already seen directly post CSDS (Supplementary Figure 1). For assessment of motor learning abilities, all mice were trained on the accelerating rotarod for 15 consecutive trials on day three post CSDS. While stress susceptible mice did not master to stay on the rod, resilient mice showed a similar learning curve as controls [maximum time: *F*_(2_,_20)_ = 27.72, *P* < 0.0001, one-way ANOVA with Tukey’s *post-hoc* test, Ctrl *n* = 10, Res *n* = 6, Sus *n* = 7 mice; Figure 1F]. This result confirmed a stress-induced change in motor skill learning in these mice, dependent on individual stress vulnerability.

### Chronic social defeat stress alters cell density in interneuronal networks in the primary motor cortex

Five weeks after CSDS, brains were collected for analysis of long-term interneuronal changes in the primary motor cortex. The density of PV positive (PV+) cells was reduced in the primary motor cortex in susceptible mice compared to controls [*F*_(2_,_20)_ = 16.14, *P* < 0.0001, one-way ANOVA with Tukey’s *post-hoc* test, Ctrl *n* = 10, Res *n* = 6, Sus *n* = 7 mice], but not in resilient mice (Figures 2A,D). The stress score was not predictive of the PV+ cell density in the controls but predicted it in the CSDS group [Ctrl: *R*^2^ = 0.054, *F*_(1_,_8)_ = 0.458, *P* = 0.518, CSDS: *R*^2^ = 0.416, *F*_(1_,_11)_ = 7.822, *P* = 0.017, simple linear regression, Figure 2B]. Layer-wise analysis of the cortex revealed, that the group effect was driven by changes in PV+ cell density of layer V, which showed a significant difference between susceptible mice and both the control and resilient group [stress *F*_(2_,_80)_ = 6.953, *P* = 0.002, layer *F*_(3_,_80)_ = 212.6, *P* < 0.0001, interaction *F*_(6_,_80)_ = 2.217, *P* = 0.05, two-way ANOVA with Tukey’s *post-hoc* test, Ctrl *n* = 10, Res *n* = 6, Sus *n* = 7 mice; Figures 2C,D].

The analysis of the SST positive (SST+) cells revealed a significantly reduced density in the primary motor cortex of susceptible mice compared to both control and resilient mice [*F*_(2_,_20)_ = 6.664, *P* = 0.006, one-way ANOVA with Tukey’s *post-hoc* test, Ctrl *n* = 10, Res *n* = 6, Sus *n* = 7 mice; Figures 2E,H]. The individual stress score was able to predict the density of SST+ cells in stressed but not in control mice [Ctrl: *R*^2^ = 0.167, *F*_(1_,_8)_ = 1.608, *P* = 0.240, CSDS: *R*^2^ = 0.417, *F*_(1_,_11)_ = 7.851, *P* = 0.017, simple linear regression; Figure 2F]. Group differences were confirmed specifically for cortical layers II/III and V [stress *F*_(2_,_80)_ = 14.60, *P* < 0.0001, layer *F*_(3_,_80)_ = 31.69, *P* < 0.0001, interaction *F*_(6_,_80)_ = 1.222, *P* = 0.304, two-way ANOVA with Tukey’s *post-hoc* test, Ctrl *n* = 10, Res *n* = 6, Sus *n* = 7 mice; Figures 2G,H].

### Chronic social defeat stress alters morphological properties of motor cortical interneurons

We next sought to dissect qualitative changes in the networks of PV+ and SST+ cells of the primary motor cortex. PV+ cell size was reduced in susceptible mice compared to resilient individuals (H_2_ = 9.488, *P* = 0.009, Kruskal-Wallis test with Dunn’s *post-hoc* test, Ctrl *n* = 1963 cells/10 mice, Res *n* = 1093 cells/6 mice, Sus *n* = 1109 cells/7 mice; Figure 3A). This effect was driven by layers II/III and V (layer II/III H_2_ = 6.151, *P* = 0.046, layer V H_2_ = 9.369, *P* = 0.009, layer VI H_2_ = 0.438, *P* = 0.804, Kruskal-Wallis tests with Dunn’s *post-hoc* test, Figures 3B,C), while layer VI was not affected (Supplementary Figure 3A). For the SST+ cell population, no significant effect of CSDS in M1 overall (H_2_ = 1.717, *P* = 0.424, Kruskal-Wallis test, Ctrl *n* = 877 cells/10 mice, Res *n* = 502 cells/6 mice, Sus *n* = 331 cells/7 mice; Figure 3D) or its layers (Supplementary Figures 3B–D) was detected.

The integrated density of PV+ cells was significantly altered in layers II/III and VI but not in layer V of M1: in layer II/III, the susceptible group had significantly lower values compared to controls while in layer VI values in the resilient group were reduced compared to both controls and susceptible mice (layer II/III H_2_ = 7.844, *P* = 0.020, layer V H_2_ = 4.904, *P* = 0.086, layer VI H_2_ = 24.89, *P* < 0.0001, Kruskal-Wallis tests with Dunn’s *post-hoc* test when applicable Figures 3F–H). For SST+ cells, significant changes of integrated staining density were restricted to layer VI with a decrease in susceptible compared to resilient mice (H_2_ = 7.496, *P* = 0.024, Kruskal-Wallis tests with Dunn’s *post-hoc* test; Figure 3I), which exhibited a slight density increase. No significant alterations were detected in layers II/III and V (Supplementary Figures 3E,F).

## Discussion

This study found evidence of long-term affection of the GABAergic interneural network of the primary motor cortex by CSDS, dependent on individual stress vulnerability.

### Stress vulnerability determined by individual burden is linked to motor learning

We used three different behavioral tests as established previously (6) to determine individual stress burden and to classify stressed animals as susceptible or resilient. We successfully developed this approach further and transformed the outcomes into a stress score, which supports a more nuanced characterization of the animals even within stress phenotypes. As demonstrated previously, a lack of correlation between the different behavioral tests occurred (6, 26), which is an important aspect to consider since the majority of studies use the SAT as the main criterion to determine stress vulnerability (5, 27). Behavior can be influenced differently by chronic stress, leading to a heterogeneous range of responses, with variable strength and permanency (27–29). In our study, nest building behavior was found to be strongly altered by chronic stress still 2.5 weeks after CSDS, marking a persistence of stress induced symptoms in susceptible mice as demonstrated in our previous work (6). Our multimodal behavioral classification using the stress score was further validated by differences in weight development and diurnal CORT release between the two stress phenotypes. Previous studies state that a typical response to stress in rodents is decreased food intake, adiposity, and body weight, due to the appearance of anhedonic behaviors and loss of interest in palatable foods (30, 31). We did not find signs of anhedonia in the SPT, or significant weight loss in susceptible mice compared to controls. On the contrary, the resilient group exhibited a prompt increase of body weight, setting them apart from the susceptible mice. Studies using CSDS usually report weight loss in susceptible mice while resilient mice have been demonstrated to either lose or gain (26, 32). As a limitation, food intake was not measured during our experiment. Glucocorticoids modulate feeding behavior but also liquid retention and can thereby promote weight gain (31, 33). Stress activates the HPA axis in order to secure homeostasis and adaptation both centrally and peripherally (5, 30, 34, 35). In our study, elevation of CORT levels was also observed solely in stress-resilient mice, which is in line with our previous data (6). Together, stress-induced HPA axis activation in resilient mice likely promotes better adaptation to chronic stress (5, 6, 30, 34). Again, we could demonstrate that motor learning on the rotarod remained intact in the resilient group whereas susceptible mice failed as shown before (6). This confirmation of the vulnerability-dependent motor learning pattern is important as the resilient phenotype was previously also accompanied by faster recovery of stress-induced spine loss of principal neurons, stability of learning-induced spine formation, and lacked the microglial and astrocytic activation in the motor cortex of susceptible mice 5 weeks post-stress (6). Although no study including ours investigated gross motor learning in such a late post-stress phase, Mizoguchi and colleagues found impaired learning on the rotarod in rats 10 days after chronic stress (36).

### Interneuronal networks in M1 respond chronically to stress depending on individual vulnerability

GABAergic interneurons control (dys) regulated excitatory output and spine density of principal neurons, which in turn can influence glial activation (11). Thus, after confirmation of the behavioral and motor functional phenotypes we proceeded to analyze the two dominant populations of GABAergic interneurons, PV+ and SST+ cells, 5 weeks post-stress. We observed an overall quantitative reduction of PV+ and SST+ interneuronal networks, which was limited to stress-susceptible individuals. Several studies have investigated the effects of chronic stressors in limbic or prefrontal cortical areas of the brain, showing a decrease of GABAergic interneurons in stress-susceptible mice, especially for PV+ and SST+ interneurons (14, 15). As mentioned, the motor cortex has rarely been investigated for such stress-induced effects before. One study in rats used M1 as a control region and did not find a change in the density of GABAergic interneurons when analyzing the entire cortex area (15). These results, contradictory to ours, could be explained by several factors, limiting comparability: use of a different (non-social) stress model, different classification of stress vulnerability, and use of a different animal species according to other studies (37, 38). Moreover, we show in our data that subtle changes in interneuronal networks could potentially be revealed best by layer-wise analysis. This also takes into consideration the layer-dependent distribution and function of GABAergic interneurons. In our previous work, astrogliosis was restricted to layer I and II/III and not found in layer V of M1 of susceptible mice (6). This is in line with the now reported reduction of PV+ cells in layer V, which in turn could lead to disinhibition and glutamatergic excess in superficial layers which can lead to astrogliosis and neuronal damage (39). Consistently, we had previously found upregulation of glutamatergic proteins in the proteome analysis of the cerebrospinal fluid in the same chronic phase 5 weeks post-stress (6).

SST + interneurons can control excitatory output and spine density of pyramidal cells by reaching out into the upper layers of the cortex and playing a pivotal role in the formation and stabilization of dendritic spines upon motor learning (20, 40). The long-term marked alteration of layer V and II/III SST+ cell density we report now fits well to the protracted recovery of spine density in susceptible mice and the impaired spine dynamics we also saw recently (6). Conclusively, another study reported a reversal of drug-induced motor cortical spine loss and impaired motor learning by activation of SST+ interneurons (41). Generally, lack of SST+ signaling underlies a depressed and anxious phenotype in mice and humans (12, 42). Although these affective behaviors are not directly linked to the motor cortex, stress burden early after CSDS was predictive of later SST+ and PV+ cell density in M1 in our study, adding new insights from an underestimated brain region regarding the stress/behavior relationship.

Changes in cell function but also decreased detectability of interneurons might be linked to alterations in morphology. Hence, we investigated soma size and staining intensity of the PV and SST markers, which can correlate with protein expression (43). PV+ cell size was altered between resilient and susceptible mice through slight up- and downregulation, respectively. Although the size reduction was not significant compared to controls, it cannot be completely ruled out that part of the decrease in cell density in susceptible mice is caused by a reduced detectability of cells slightly smaller than our determined threshold. Since the lower size threshold was carefully established using spatial correlation with nuclear DAPI staining we do not consider this as a relevant confounder. Reduced soma size could be linked to a shift in certain PV+ subpopulations (19) caused by a selective loss in susceptible mice. This could be assessed in future studies by application of co-staining techniques, high-resolution imaging of cell morphology, and electrophysiological characterization, as over 20 different subtypes of GABAergic interneurons in the cortex have been distinguished to date, which differ on the markers they express but also on firing patterns, morphology, and regional distribution (18, 19, 44).

**FIGURE 4:**
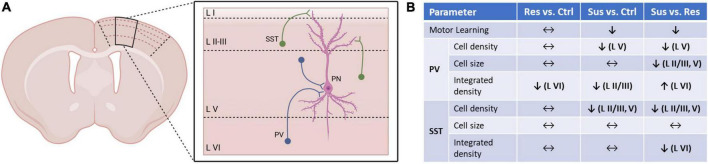
Summary of the quantitative and qualitative changes in interneuronal networks of M1 and its layers dependent on individual stress vulnerability. **(A)** Schematic network of parvalbumin positive (PV+) and somatostatin positive (SST+) interneuron populations investigated in this study and the principal neurons (PN) of layer V of the primary motor cortex (black outline on coronal section) previously found to be affected by CSDS (6). **(B)** Comparisons between the stress phenotypes (Res, Sus) and controls (Ctrl), and between the stress phenotypes to dissect the implication of stress vulnerability for motor cortical interneurons also in relation to motor learning abilities on the rotarod.

Quantity of the PV and SST staining expressed in the integrated density was differentially altered depending on stress phenotype, cortical layer, and the marker itself. These effects were dissociated from changes in cell density or size and point to a chronic qualitative change of interneurons predominantly in layer VI. Of note, this was detected specifically in layer VI from resilient animals, a layer which had not been affected by changes in density or cell size and highlights subtle network changes induced by stress in presumably “healthy” subjects with unchanged cell numbers. Although the layer-wise connections between principal and interneuronal networks (exemplary depiction in Figure 4A) are still not completely understood (11, 40), layer VI exerts excitatory output into layer V (40) and thus its disinhibition can facilitate stress-induced hyperexcitability in the superficial layers of M1 in susceptible mice. Viewing quantitative and qualitative changes together (Figure 4B) a dissociation between reduced cell density and integrated density is notable. Hence, the reduction of detected PV+ and SST+ cells in layers II/III and V is unlikely to be caused by a general downshift of immunoreactivity. Furthermore, this is the only technique allowing for high spatial resolution and thus layer-wise analysis, which would be masked by methods using whole tissue samples.

Together with our previous data of synaptic loss, glial activation, and CSF alterations (6), the disturbance of the GABAergic interneuron network reflects another indicator of stress-induced hyperexcitability in the motor cortex, which could underly impaired motor learning. Glutamatergic excess, as mentioned above, generally promotes neurotoxicity which could be underlying cell loss and degeneration in the interneuronal networks of M1. Reduction of PV and SST has been linked to proinflammatory and neurodegenerative states (43, 45). We previously found signs of both states in M1 and the cerebrospinal fluid of mice more than 5 weeks post CSDS (6). This indicates persistent stress-induced negative effects on neuronal and non-neuronal cell populations of M1 that are likely propelling each other. Our study further highlights the importance of understanding long-term neurobiological outcomes of chronically stressed individuals considering the chronic timecourse of stress related neuropsychiatric disorders.

## Data availability statement

The datasets generated during and/or analyzed during the current study are available from the corresponding author on reasonable request.

## Ethics statement

The animal study was reviewed and approved by the Local Committee for Animal Health, LANUV NRW.

## Author contributions

AKG: conceptualization and funding acquisition. MS and AKG: methodology and investigation and writing—original draft. MS, VS, and AKG: writing—review and editing. VS and AKG: resources and supervision. All authors contributed to the final version of the manuscript.

## Conflict of interest

The authors declare that the research was conducted in the absence of any commercial or financial relationships that could be construed as a potential conflict of interest.

## Publisher’s note

All claims expressed in this article are solely those of the authors and do not necessarily represent those of their affiliated organizations, or those of the publisher, the editors and the reviewers. Any product that may be evaluated in this article, or claim that may be made by its manufacturer, is not guaranteed or endorsed by the publisher.
