# Supplementary material for: Long-term changes of parvalbumin- and somatostatin-positive interneurons of the primary motor cortex after chronic social defeat stress depend on individual stress-vulnerability
Source: Front Psychiatry. 2022 Jul 28;13:946719. doi: 10.3389/fpsyt.2022.946719 (PMC9366473; doi:10.3389/fpsyt.2022.946719)
Supplement: Supplementary file 1 [file Data_Sheet_1.docx]

Supplemental Figures

**
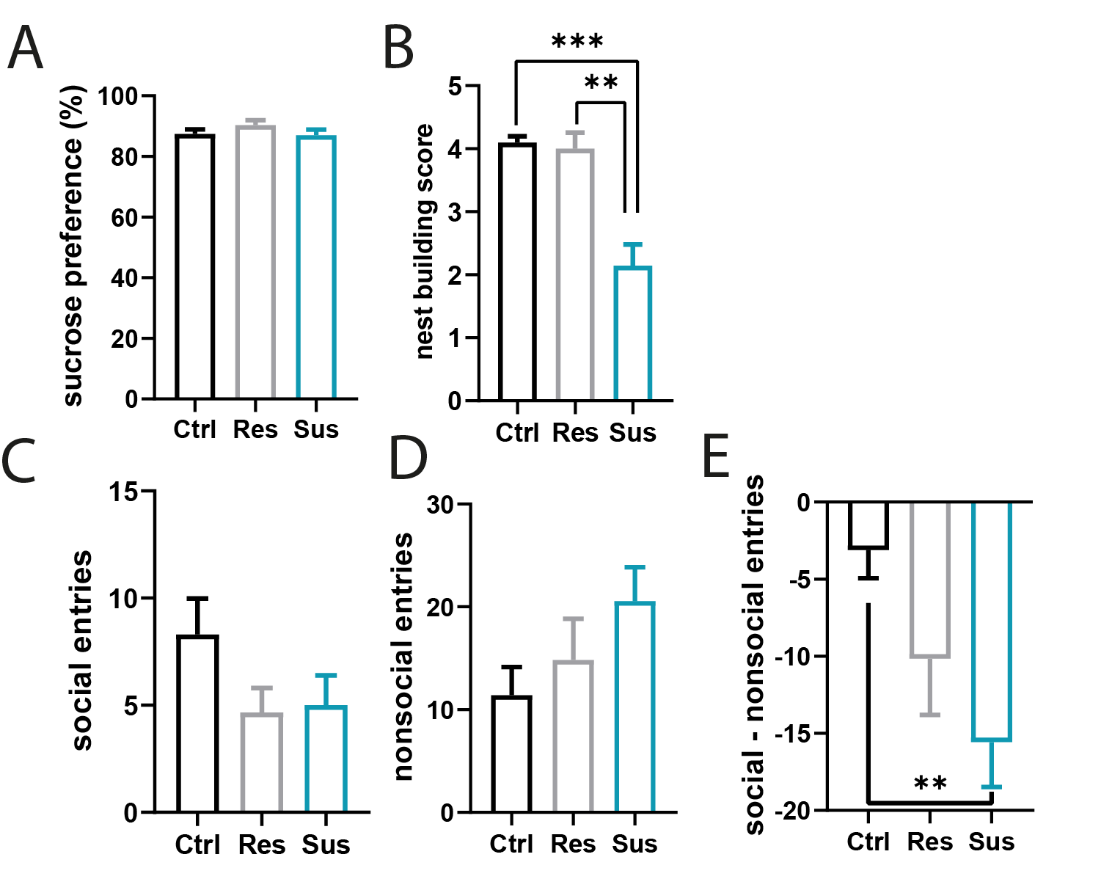
**

**SFigure 1. Behavioral assessment underlying the stress score.** Behavior was measured within 3 days after the last CSDS session. (A) Sucrose preference did not differ significantly between controls and stressed mice (F_2,20_ = 1.023, P = 0.378, one-way ANOVA, Ctrl n = 10, Res n = 6, Sus n = 7 mice), while the (B) nest building score in the nestlet shredding test was severely reduced in the susceptible group compared to control and resilient mice (H_2_ = 16.38, P = 0.0003, Kruskal-Wallis test with Dunn’s post-hoc test, Ctrl n = 10, Res n = 6, Sus n = 7 mice). In the social avoidance test, number of entries into the interaction zone with and without presence of a social partner were not significantly different but their difference revealed a significant drop in socializing attempts in susceptible individuals (social entries: F_2,20_ = 1.805, P = 0.190, nonsocial entries: F_2,20_ = 2.167, P = 0.141, social – nonsocial entries: F_2,20_ = 6.201, P = 0.008, one-way ANOVA with Tukey’s post-hoc test when applicable, Ctrl n = 10, Res n = 6, Sus n = 7 mice). **P < 0.01, ***P < 0.001. Results are shown as mean ± SEM.

**
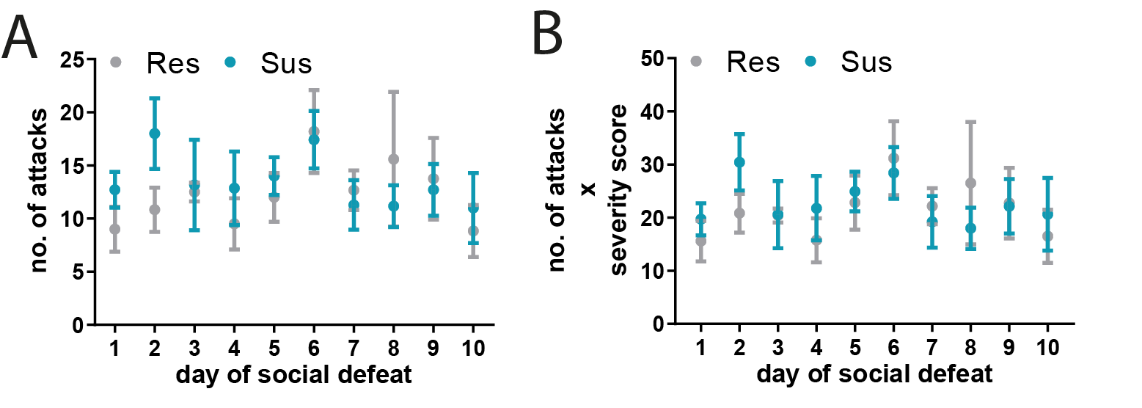
**

**SFigure 2. Quantitative and qualitative analysis of CSDS attacks.** Differences in CSDS attack quantity and quality as a cause for the two stress phenotypes could be ruled out as (A) daily number of attacks (time F_4.308,48.83_ = 0.1.144, P = 0.348; phenotype F_1,102_ = 0.764, P = 0.384; interaction F_9,102_ = 0.621, P = 0.777; RM ANOVA mixed model) and (B) cumulative severity of attacks (F_4.219,47.82_ = 0.960, P = 0.441; phenotype F_1,102_ = 0.222, P = 0.638; interaction F_9,102_ = 0.471, P = 0.891; RM ANOVA mixed model) did not differ between resilient and susceptible groups. Res n = 6, susceptible n = 7 mice. Results are shown as mean ± SEM.


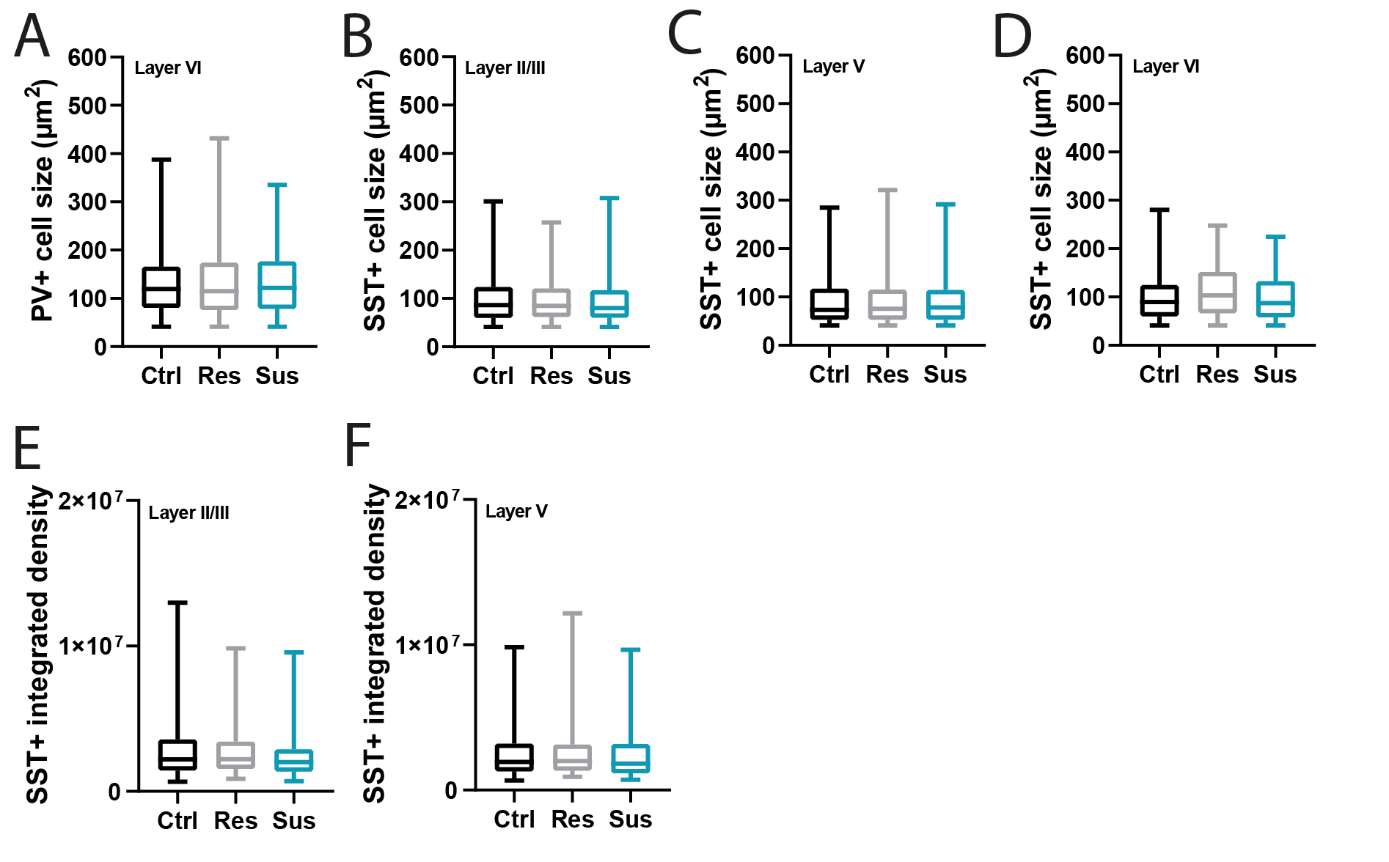
**SFigure 3. Cell size and integrated density of PV+ and SST+ cells in different layers of M1.** (A) PV+ cell size of cortical layer VI did not differ between controls and the stress phenotypes (H_2_ = 0.4375, P = 0.804, Kruskal-Wallis test). (B-D) No significant effect of CSDS was detected in the SST+ cell population of layer II/III, V, and VI (layer II/III H_2_ = 0.726, P = 0.696, layer V H_2_ = 0.1519, P = 0.927, layer VI H_2_ = 5.553, P = 0.062, Kruskal-Wallis test). (E) SST+ fluorescence intensity was not changed by CSDS in cortical layers II/III and V (layer II/III H_2_ = 3.684, P = 0.162, layer V H_2_ = 1.624, P = 0.444, Kruskal-Wallis tests). Results are shown as median and ranges.
